# Cytokine Response Profiles of School-Aged Children Infected with Schistosomiasis before and after Praziquantel Treatment

**DOI:** 10.1155/2021/6678981

**Published:** 2021-06-17

**Authors:** Edward Okonjo, Dorcas Yole, Dorington Ogoyi

**Affiliations:** ^1^Department of Applied and Technical Biology, Technical University of Kenya, P.O. Box 52482-00200, Nairobi, Kenya; ^2^National Biosafety Authority, P. O Box 28251-00100, Nairobi, Kenya

## Abstract

Schistosomiasis is a parasitic disease that affects millions of people in 78 countries globally. Children under the age of 14, who have the chronic disease may suffer from anemia and malnutrition that contribute to lost days at school and pervasive learning disabilities. The infection is prevalent in Kenya, especially in endemic areas, contributing to significant morbidity. The cellular response pattern is associated with both the acute and chronic phases of the disease, in which cytokines play a critical role. The objective of this study was to evaluate the cytokine profiles of IL-4, IL-2, IL-10, IL-5, IFN-*γ,* and TNF in serum samples of infected school-aged children by using flow cytometry before and after treatment. The analysis indicated a shift in the expression of the cytokines after treatment with all the cytokines being downregulated, except TNF. There was a general trend of decrease in the expression of the cytokines at six and twelve weeks after treatment as compared to the pretreatment levels. There were statistically significant differences in the expression in IL-2 (*P*=0.001^*∗∗*^), IL-4 (*P*=0.033^*∗*^), IL-10 (*P*=0.001^*∗∗∗*^), IFN-*γ* (*P*=0.023^*∗*^), and IL-5 (*P*=0.0001^*∗∗∗*^), except in TNF (*P*=0.095). The reduction in the cytokine levels can be directly related to the influence of the drug praziquantel, modulating the cytokine response by elimination of adult worms, decline in parasitic load, and reduction of morbidity. Therefore, cytokine response is directly related with the influence of treatment in the variation of the immune response.

## 1. Introduction

Schistosomiasis is a chronic severe illness with estimates from the World Health Organization (WHO) showing that at least 290.8 million people required preventive treatment in 2018, in which more than 97.2 million people were reported to have been treated [1]. In endemic areas, most infected people have the asymptomatic intestinal form detected during the chronic phase of infection [2].

The infection induces both cellular and humoral responses that correlate with the acute and chronic form of the disease. Both T helper 1 (Th1) and T helper 2 (Th2) responses are induced in response to the infection [3]. Th1 and Th2 responses are crucial in understanding the role of cytokines in the immune response to infectious agents [4, 5]. Studies have shown strong associations between schistosoma infection and schistosoma-induced cytokine profiles [6]. Th1 cells that produce IL-2, IFN-*γ*, TNF-*α,* and TNF-*β* evoke cell-mediated immunity and phagocyte-dependent inflammation while Th2 cells that produce IL-5, IL-4, IL-6, IL-10, and IL-13 induce a strong antibody response that comprises the IgE class and eosinophil accumulation, but it inhibits several functions of phagocytic cells [7]. Corrêa-Oliveira et al. [8] speculated that IL-10 is important in regulating the human immune response to *Schistosoma mansoni* infection and that it is the main cytokine that plays part in the control of morbidity.

In mouse models of *S. mansoni*, the Th1 response correlated with partial protection, while the Th2 response correlated with increased susceptibility and severe pathology. [9–11]. However, human studies have shown that, during infection, the immune response advances through at least three phases. Initially, in the acute phase in which the host harbors migrating immature parasites, the main response is Th1 with the production of TNF, IL-1, and IL-6 [3]. This response reduces the pathology associated with the inflammatory reaction as a result of the infection; however, cellular responses may still not be sufficient to eliminate the parasite [12]. In phase 2, as the parasites mature and reproduce with the production of eggs, the Th1 response is reduced and a strong Th2 response develops, induced mostly by egg antigens. In the chronic phase of infection, forming phase 3, the Th2 response is moderated and granulomas, which form around newly deposited eggs, become smaller than they were earlier during the infection [3].

Schistosomes are known to downregulate host immune responses and to induce a modified Th2 response [13]. Helminth infections are typically linked with Th2-dependent responses leading to IgE production and eosinophilia. For schistosomiasis, Th2 responses with the production of IgE antibodies against a restricted range of adult worm antigens are associated with protection against reinfection after chemotherapy. Individuals in whom Th2 responses against egg antigens predominate show less severe egg-associated morbidity than those where Th1 responses predominate. Consequently, Th2-mediated responses are thought to be selectively advantageous to the human host in *S. mansoni* infection and perhaps other helminth infections [13]. Cellular and humoral immune responses contribute to protection. Treating schistosome-infected individuals has been known to alter the schistosome-specific cellular and humoral immune responses. It is important that the cellular response patterns before and after treatment are understood as a basis for understanding the importance of the immunological aspects of therapeutic interventions. School-aged children are at a higher risk due to reinfection and an approach to understanding their immunological response mechanisms after treatment provides a new insight in schistosomiasis control. The study sought to evaluate cytokine (IL-2, IL-4, IL-5, IL-10, TNF, and IFN-*γ*) profiles in the serum of children infected with schistosomiasis before and after treatment with praziquantel (PZQ).

## 2. Methodology

### 2.1. Ethics Statement

This longitudinal study was part of a larger study on schistosomiasis transmission dynamics in Mwea Irrigation Scheme in Kirinyaga County. The children recruited into the study were followed before treatment, six weeks after treatment, and twelve weeks after treatment. Ethical approval was obtained from the Kenyatta National Hospital/University of Nairobi Ethical Review Board no. P111/03/2015. Informed and written consent was obtained from the legal guardians of all the children from Mianya Primary School participating in the study before inclusion into the study. The inclusion criteria included children-aged between 7 and 14 years who were positive for *S. mansoni* and who had no severe medical condition or existing disease. Children who had PZQ treatment in the last 6 months before the study were excluded. Sample size determination was based on the following formula as per the WHO (2013) [14] guidelines:

(1) $$\begin{array}{c} \text{No. of children to be screened}=\frac{\text{No. of infected children}}{\text{compliance rate}\times \text{prevalence}}. \end{array}$$

The compliance rate is the percentage of the children identified as positive at baseline who will provide a stool and blood specimen at follow-up.

### 2.2. Sample Collection (Stool and Blood)

A container for faecal sample was given to each pupil. The faecal specimens were analyzed with the Kato-Katz method [15] and quantification was done as eggs per gram of faecal sample (EPG) classified as either light (≤100 EPG), moderate (≥101–399 EPG), or heavy (≥400 EPG). Each slide preparation was done in duplicate and an average of the EPG was done.

All pupils who tested positive for *S. mansoni* had their blood samples taken. Three ml of venous blood sample from each pupil was collected before treatment. The blood was collected in BD Vacutainer blood collection tubes by qualified phlebotomists from Kimbimbi Subcounty Hospital and transported to the Division of Vector Borne Disease (DVBD) laboratory in a cool box for serum preparation. Serum was prepared by leaving the samples overnight at 4^o^C. The serum was aliquoted and stored at −20^o^C.

### 2.3. Treatment of Infected Children

All the positive cases were treated with PZQ (Prazitel®, Cosmos Ltd.) A qualified nurse administered the PZQ according to the prescribed dose of 40 mg/kg weight using a WHO dose pole. The drug was administered orally as a single dose. The procedure for stool and blood collection was repeated after six and twelve weeks on the same pupils.

### 2.4. Cytokine Assay Procedure

Cytokine analysis was carried out using the BD Cytometric Bead Array (CBA) Human Th1/Th2 Cytokine Kit (catalogue no. 550749) from Becton Dickson International (Belgium). The protocol used was contained in the Th1/Th2 human cytokine kit instruction manual. This analysis was carried out at the Kenya Medical Research Institute (KEMRI), Centre for Biotechnology Research and Development (CBRD), using a BD FACSCalibur, Becton Dickinson (serial number E97500228) flow cytometer. The Th1/Th2 standards were provided in the test kit and they were prepared alongside the test serum samples. The standards were run on the flow cytometer from the lowest to the highest concentration followed by the test samples. The intensity of fluorescence varied according to the cytokine expressed and the complexes were measured and detected after acquiring sample readings. The Flow Cytometry Data Analysis and Processing Software (FCAP Array™ Software) was used to generate results in graphical and tabular format.

### 2.5. Cytokine Data Analysis

Mean fluorescent intensity (MFI) data from the various treatments under each cytokine were subjected to one-way ANOVA to determine significant differences at pretreatment, 6 weeks after treatment, and 12 weeks after treatment. Probability/significance level used in all ANOVA analyses was at *P* < 0.05. A Tukey's multiple comparison post hoc analysis was performed to show the statistical differences in between the groups. Statistical analysis was performed by the use of the GraphPad Prism 5 software.

## 3. Results

### 3.1. Parasitological Data

The mean percentage baseline prevalence of infection before treatment was 68%. According to the infection intensity, heavy infections accounted for 42%, moderate infections 30%, and light infections 28%.

### 3.2. Prevalence at 6 and 12 Weeks

The prevalence of infection six weeks after treatment was 20% with 33% light infections, 42% moderate infection, and 25% heavy infection. At 12 weeks after treatment, the overall prevalence rate was 50% with 61% light infections, 31% moderate infection, and 7% heavy infection (Table 1).

**Figure 1:**
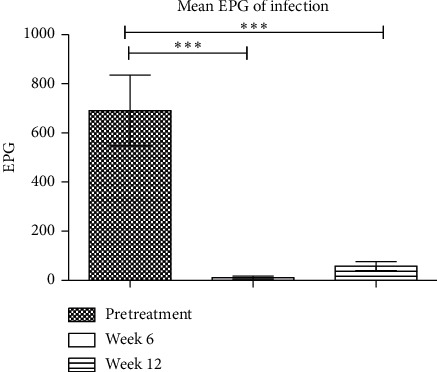
Mean EPG of infection.

Mean EPG was significantly reduced at six and twelve weeks after treatment, *P*=0.001^*∗∗∗*^ (Figure 1).

### 3.3. Cytokine Profiles

Evaluation of cytokine expression intensities before and after treatment showed a considerable shift in the levels of individual cytokines expressed. Generally, from the mean MFI values, TNF (6.65), IL-10 (4.56), and IL-5 (5.22) expressed a higher concentration in the individuals both in pretreatment levels and at 6 and 12 weeks after treatment as compared to the others.

### 3.4. Interferon-*γ* (IFN-*γ*)

**Figure 2:**
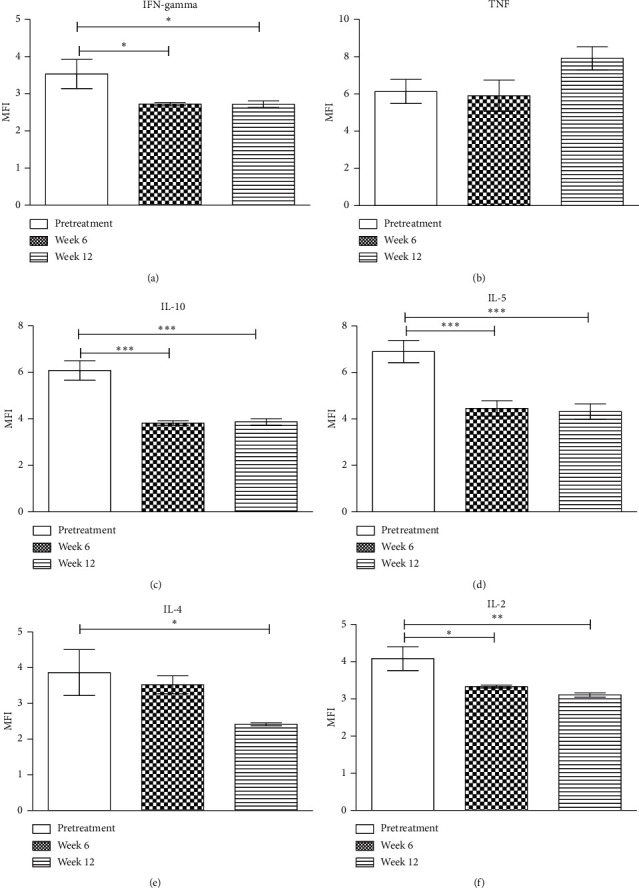
(a)–(f) Cytokine profiles showing changes in expression of individual cytokines before treatment, 6 weeks after treatment, and 12 weeks after treatment. ^∗∗∗^Highly significant. ^∗∗^Moderately significant. ^∗^Slightly significant.

There was a decrease in IFN-*γ* expression from the pretreatment levels, *P*=0.023^*∗*^. There were statistically significant MFI differences between pretreatment and at 6 weeks after treatment. Similarly, there were statistically significant MFI differences between pretreatment and at 12 weeks after treatment. However, there were no statistically significant MFI differences between 6 weeks and 12 weeks after treatment in IFN-*γ* (Figure 2(a)).

### 3.5. Tumor Necrosis Factor (TNF)

From the values obtained in TNF, there was a very slight reduction in expression at 6 weeks after treatment as compared to pretreatment levels. There was an increase at 12 weeks after treatment (Figure 2(b)). However, statistically these differences were not significant, *P*=0.095.

### 3.6. Interleukin-10 (IL-10)

There was a drop in IL-10 expression at six weeks and 12 weeks after treatment as compared to pretreatment levels (Figure 2(c)). These differences were statistically significant (*P*=0.001^*∗∗∗*^) at 6 and 12 weeks after treatment. There was no statistical difference between 6 weeks and 12 weeks after treatment.

### 3.7. Interleukin-5 (IL-5)

There was a drop in IL-5 expression at 6 and 12 weeks after treatment as compared to pretreatment level. There were statistical differences between pretreatment and 6 weeks after treatment, *P*=0.0001^*∗∗∗*^. However, there was no statistical difference between 6 weeks and 12 weeks after treatment (Figure 2(d)).

### 3.8. Interleukin-4 (IL-4)

There was a decrease in IL-4 expression between the pretreatment levels and 6 and 12 weeks after treatment (Figure 2(e)) that was statistically significant, *P*=0.033^*∗*^. There were statistical differences between pretreatment and 12 weeks after treatment. However, no significant differences between pretreatment IL-4 levels and 6 weeks after treatment and also between 6 weeks and 12 weeks after treatment.

### 3.9. Interleukin-2 (IL-2)

IL-2 production decreased both at week 6 and 12 after treatment in comparison with pretreatment levels (Figure 2(f)). These differences were statistically significant, *P*=0.001^*∗∗*^. There were statistically significant differences between pretreatment and 6 weeks after treatment and pretreatment and 12 weeks after treatment. There was no statistical difference between 6 weeks and 12 weeks after treatment.

The summary of the mean MFI differences for each cytokine is give below at 95% CI (Table 2)

## 4. Discussion

Cytokines being key immunological messengers are critical in the regulation of most of the biological responses in the immune system and in particular for this study in the immune response to schistosomiasis. The cytokines studied were from the CD4+ T cell subpopulations of Th1 (IFN-*γ*, TNF, and IL-2) and Th2 (IL-4, IL-5, and IL-10) subsets. The Th1 subset is linked with cellular responses, while Th2 subset, triggered by egg-derived antigens is primarily involved in B cell and antibody production [16, 17].

In this study, there was a shift in levels of IL-4, IL-5, IL-10, IL-2, TNF, and IFN-*γ* after treatment as compared to pretreatment levels. All the cytokines except TNF showed a decline in their expression at six and twelve weeks after treatment. Administration of PZQ effectively reduced the parasite load and this directly influenced the expression of these cytokines. Similar studies in a baboon model showed that cytokines peaked during the acute infection, declined with chronic infection, and became undetectable after treatment [18]. However, in the current study, they declined with treatment of PZQ at six weeks but their levels never declined further after twelve weeks possibly due to high reinfection rates of the children which then alter the immune response. Before treatment, the children in this study had high parasite load especially with the moderate and heavy infection. Antiparasitic chemotherapy has been shown to reverse the impairment of cell-mediated immune responses at the cytokine level [19]. Consequently, in the present study, treatment with PZQ had a direct influence in the expression of the cytokines as a result of the decline in parasitic load and the immune response elicited.

IFN-*γ* plays a key role as an activator of macrophages and inducer of Class II major histocompatibility complex (MHC) molecule expression and is produced once antigen-specific immunity develops [20] and this is important to acquire resistance to schistosomes. In this study, IFN-*γ* was present at pretreatment although not at very high levels, but its expression was significantly reduced at six and twelve weeks after treatment. Following treatment, there was a reduction in worm loads and activation of the macrophages together with the Th1 response, which consequently led to the reduction in the expression of IFN-*γ*. Early stage of the disease is related to a significant IFN-*γ* response and is a major downregulator of fibrosis which is reduced when the worms are eliminated [21]. This is also true for this study in school-aged children which shows that treatment with consequent reduction in worm loads leads to a reduction in IFN-*γ*. It is only during the acute (initial) phase of the infection and in endemic normal individuals that the IFN-*γ* levels are normally elevated [22].

TNF is involved in systemic inflammation and is one of the cytokines that comprise the acute phase reaction and is associated with proinflammatory and profibrogenic effects [22], but it could also be protective [23]. In this study, there was a slight decrease in TNF levels in week six and then increase by week twelve though these were not statistically significant. This was related very positively to the children who had moderate and high infections during these times. TNF-*α* has been known to be significantly high in individuals with a higher risk of periportal fibrosis [22]. Considering that it is actually related with *S. mansoni* parasite burden, it may be used as a biomarker for the development of liver pathology in schistosomiasis [24]. For this study, it may be so in children who have developed fibrosis as a consequence of prolonged infection. However, this cannot be concluded because other factors may have influenced this outcome.

IL-10 is a cytokine with multiple functions in immunoregulation and inflammation. It downregulates the expression of Th1 cytokines such as IFN-*γ* and TNF-*α*, MHC Class II antigens, and costimulatory molecules on macrophages [25]. It also improves B cell survival, proliferation, and antibody production. In this study, IL-10 expression was elevated at pretreatment and reduced at week six and week twelve. Therefore, the fact that it was present in high quantity at pretreatment may have influenced the progression of the infection by preventing excessive inflammation in early stages. It plays a major part in suppressing immune and inflammatory responses and this contributes directly to the response in both early and chronic infections [12]. Conversely, IL-10 has got a direct influence on IFN-*γ* which has been known to be specifically associated with protection against fibrosis. The production of IFN-*γ* depends on the relative concentration of IL-10, hence it is thought that IL-10 may be functionally important in the development of morbidity in schistosomiasis and disease progression [26]. This concurs with the present study in that its expression of IFN-*γ* in the infected children may have been hindered because of the cross-regulatory effect of IL-10 which was present in high quantities before treatment.

Similar findings have suggested that chemotherapy contributes to the influence of IL-10 on IFN-*γ* either as a consequence of the elimination of parasite eggs or through the generation of large number of new antigens by dead and/or damaged parasites [27]. This study showed that Th1 and Th2 cell responses cross-regulate each other. Thus, IFN-*γ* downregulates Th2 cell development, while IL-4 and IL-10 antagonize Th1 cell differentiation [28].

IL-5 is an important cytokine because it regulates eosinophils whose levels in humans are associated with resistance and is important at the onset of infection. The role of IL-5 in acute experimental schistosomiasis shows that it is required for blood and tissue eosinophilia but not for granuloma formation. It is a key constituent in the tissue response against challenge infection. The later stages of differentiation and maturation of most of the eosinophil population are controlled by the cytokine IL-5 [29].

In this study, IL-5 was reduced at six and twelve weeks after treatment. Helminth infections are associated with marked and/or persistent eosinophilia. An increase in eosinophil levels before treatment in this case was a marker for infections. IL-5 levels were elevated at pretreatment showing infection. Consequently, a reduction in the levels of expression of IL-5 at six and twelve weeks after treatment is indicative of reduced eosinophil levels in circulation because of the elimination of the parasite. In addition, findings have shown that there is an influence of IFN-*γ* on IL-5 which plays a role in regulating the uncontrolled release and the consequent elevation in circulating eosinophils. Both are involved in Th1 response and they work together in influencing the immune response and this reflects previously documented influence of IFN-*γ* on IL-5 [29].

IL-4 which marks the Th2-like response is known to stimulate B cells to selectively mature and proliferate for production of particular antibody isotypes, which could eventually be involved in antibody-dependent cell-mediated cytotoxicity (ADCC) reactions, resulting in multicellular parasite killing and preventing any further invasion [30]. In this study, IL-4 was significantly reduced at week six and twelve after treatment as compared to pretreatment levels. This is expected and important because of the changes in the acute and chronic phase of the disease which have now been controlled. Their initial expression before treatment was important in helping to mount a humoral response to the infection. However, this contrasts with a previous study [31], which showed that one month after treatment with praziquantel there was an increase in the production of IL-4 and IL-10 cytokines following SEA stimulation in circulating lymphocytes from infected patients.

IL-2 is primarily responsible for activating T and B lymphocytes and is integral to bringing about the acquired immune response, being a stimulant of T and B cell growth and maturation. For the present study, IL-2 expression was reduced after treatment and this reduction was significant. This is attributed to the fact that it had already elicited the stimulation of the T and B cells at the beginning of infection, and following treatment and elimination of parasite, T and B cell production was reduced. Similar studies [32, 33] found that there was reduction in IL-2 and IFN-*γ* after treatment.

## 5. Conclusion

Cytokine response is directly related to therapeutic intervention leading to elimination of adult worms, decline in parasitic load, and eventually reduction of morbidity. In school-aged children, their immune system is still developing and not as mature as the adults. The general decline in the amount of cytokine expression after treatment except for TNF related directly with the reduction in worm loads during the same period [34]. Additionally, heavy infections declined significantly after treatment relating directly to the influence that chemotherapy has in the expression of the Th1 and Th2 cytokine response. TNF, IL-10, and IL-5 collectively had higher concentration as compared to the others in the children both in pretreatment levels and at six and twelve weeks after treatment compared to other cytokines. High levels of IL-5 and TNF suggested that the children may be suffering from morbidity associated with *S. mansoni,* especially due to the fact that most children had heavy infection intensities before treatment. The high levels of TNF throughout the period were an indicator that the children may have had a prolonged infection and were at risk of development of pathology associated with schistosomiasis.

The results in this study are significant in understanding the modulation of the immune response due to treatment. The administration of praziquantel altered the cellular response as indicated by the downregulation of the Th1 and Th2 cytokines in sera. This in turn is important in strengthening the body's immune response mechanism, especially in school-aged children who are very prone to reinfection.

In order to fully understand the immune system response mechanism, there is need to evaluate these responses in the acute and chronic phase of the disease over a prolonged study period.

## Figures and Tables

**Table 1:** Prevalence of light, medium, and heavy schistosomiasis after PZQ treatment.

Infection intensity	Frequency, *n* (%)		
	Before	At 6 weeks	At 12 weeks
Light (<100 EPG)	21 (27.6)	4 (33)	16 (62)
Medium (100–399 EPG)	23 (30.3)	5 (42)	8 (31)
Heavy (≥400 EPG)	32 (42.1)	3 (25)	2 (7)

EPG, number of eggs per gram faecal sample.

**Table 2:** Comparison of mean MFI differences of each cytokine.

Cytokines	Pretreatment	6 weeks after treatment	12 weeks after treatment	*P* value (*P* < 0.05)
IFN-*γ*	3.53 (95% CI: 0.01384 to 1.605)	2.72 (95% CI: 0.01784 to 1.609)	2.72 (95% CI: −0.7918 to 0.7998)	0.023^*∗*^
TNF-*α*	6.13 (95% CI: −2.179 to 2.626)	5.90 (95% CI: −4.195 to 0.6108)	7.92 (95% CI: −4.418 to 0.3872)	0.095 NS
IL-10	6.08 (95% CI: 1.370 to 3.145)	3.82 (95% CI: 1.320 to 3.095)	3.87 (95% CI: −0.9378 to 0.8378)	0.001^*∗∗∗*^
IL-5	6.89 (95% CI: 1.132 to 3.756)	4.45 (95% CI: 1.263 to 3.887)	4.32 (95% CI: −1.181 to 1.443)	0.0001^*∗∗∗*^
IL-4	3.86 (95% CI: −1.014 to 1.699)	3.52 (95% CI: 0.08924 to 2.803)	2.41 (95% CI: −0.2532 to 2.460)	0.033^*∗*^
IL-2	4.08 (95% CI: 0.1094 to 1.399)	3.33 (95% CI: 0.3338 to 1.624)	3.10 (95% CI: −0.4206 to 0.8694)	0.001^*∗∗*^

^*∗∗∗*^ = highly significant. ^*∗∗*^ = moderately significant. ^*∗*^ = slightly significant. NS=not significant.

## Data Availability

The data used to support the findings of this study are available from the corresponding author upon request.
